# Molecular and Cellular Functions of the Warsaw Breakage Syndrome DNA Helicase DDX11

**DOI:** 10.3390/genes9110564

**Published:** 2018-11-21

**Authors:** Francesca M. Pisani, Ettore Napolitano, Luisa M. R. Napolitano, Silvia Onesti

**Affiliations:** 1 Istituto di Biochimica delle Proteine, Consiglio Nazionale delle Ricerche, Via P. Castellino, 111, 80131 Napoli, Italy; e.napolitano@ibp.cnr.it; 2 Elettra–Sincrotrone Trieste S.C.p.A., AREA Science Park Basovizza, 34149 Trieste, Italy; luisa.napolitano@elettra.eu

**Keywords:** genome stability maintenance, DNA replication, DNA repair/recombination, sister chromatid cohesion, DNA helicase, iron-sulphur cluster, DDX11, Warsaw breakage syndrome

## Abstract

DDX11/ChlR1 (Chl1 in yeast) is a DNA helicase involved in sister chromatid cohesion and in DNA repair pathways. The protein belongs to the family of the iron–sulphur cluster containing DNA helicases, whose deficiencies have been linked to a number of diseases affecting genome stability. Mutations of human DDX11 are indeed associated with the rare genetic disorder named Warsaw breakage syndrome, showing both chromosomal breakages and chromatid cohesion defects. Moreover, growing evidence of a potential role in oncogenesis further emphasizes the clinical relevance of DDX11. Here, we illustrate the biochemical and structural features of DDX11 and how it cooperates with multiple protein partners in the cell, acting at the interface of DNA replication/repair/recombination and sister chromatid cohesion to preserve genome stability.

## 1. Introduction

**Figure 1:**
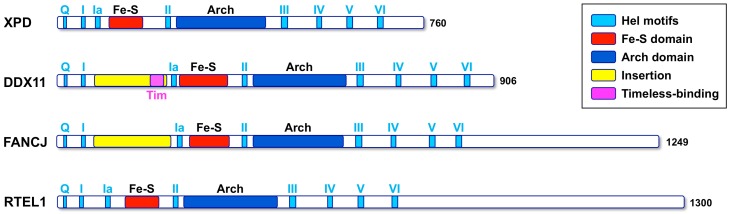
Schematic representation of the architecture of the human Fe–S DNA helicases. The colour code for the domains and motifs is shown in the inset. The conserved helicase motifs are shown in *light blue*; box I and II correspond to the canonical ATPases Walker A and Walker B motifs. The characteristic Fe–S and Arch domain are shown in *red* and *blue*, respectively. Two partially unstructured insertions (shown in *yellow*) are present in FANCJ and DDX11, between motifs I and Ia; the region in DDX11 that has been shown to interact with Timeless (in *magenta*) is located within this insertion.

DDX11 (also named ChlR1) is an ATP-dependent DNA helicase with 5′ to 3′ directionality, belonging to the super-family 2 (SF2) DNA helicases. The presence of an iron–sulphur cluster (Fe–S) domain classifies DDX11 as a member of the subgroup of Fe-S DNA helicases, which also encompasses the *Xeroderma pigmentosum* group D (XPD) protein, as the subclass prototype, FANCJ and RTEL1 (see Figure 1) [1]. All these SF2 Fe–S DNA helicases play critical functions in the maintenance of genome stability and are linked to rare genetic syndromes and cancer predisposition [2]. Autosomal recessive mutations of the *DDX11* gene are responsible for a rare cohesinopathy, named Warsaw breakage syndrome (WABS) [3].

Here, we review what is known about the roles played by human DDX11 in cellular DNA metabolism with an emphasis on the biochemical properties and structural features of this DNA helicase. Furthermore, we highlight the most recent discoveries of DDX11 functions in sister chromatid cohesion and in DNA repair and describe its medical relevance.

## 2. Discovery of Human DDX11

Human DDX11 was first discovered by Frank and Werner in a study reporting the identification of genes regulated by the keratinocyte growth factor (KGF) in keratinocytes [4]. Molecular cloning and sequencing of the corresponding full-length complementary DNA (cDNA) revealed a strong similarity with the *Saccharomyces cerevisiae CHL1* gene. Shortly after, two human cDNAs were isolated in the Lahti laboratory and characterized as having high similarity to the product of the same yeast *CHL1*/*CTF1* gene [5,6]. *CHL1*/*CTF1* was identified in a genetic screen of yeast mutants with decreased chromosome transmission fidelity (*CTF*) leading to chromosome loss (*CHL*). The corresponding Chl1/Ctf1 protein contains all the conserved sequence motifs that are characteristic of a SF2 DNA helicase [7]. Based on genomic studies, two distinct human Chl1-related (CHLR) cDNAs were found to be encoded by two highly similar (98% identity) genes that were named *CHLR1* and *CHLR2* [6]. These genes were localised to human chromosome regions 12p11 and 12p13 and were proposed to be generated by gene duplication. The same region of chromosome 12 likely underwent several duplication and translocation events, since sequences highly similar to the C-terminal portion of human *CHLR1* were identified in putative pseudogenes present in the subtelomeric regions of many human chromosomes. More recently, Costa and co-workers revisited the *CHLR1* gene duplication/translocation hypothesis and proposed that an ancestral *DDX11* gene gave rise to a novel family of genes that are characterized by a common subtelomeric location and a similar C-terminal sequence [8]. 

Studies of human *CHLR* genes revealed that they are expressed only in proliferating cells and not in serum-depleted cultured cells. Quiescent normal human fibroblasts stimulated to re-enter the cell cycle by addition of serum begin to express the CHL1-related proteins as the cells enter S phase. Affinity-purified antisera directed against ChlR1 were used to demonstrate that this protein has a nuclear localization, by indirect immunofluorescence and cell fractionation coupled to Western blot analysis [6]. Recombinant human ChlR1/DDX11 protein was purified and shown to possess an ATPase-dependent DNA unwinding activity in vitro, as described in Section 3. Conversely, the putative human ChlR2 protein (also named DDX12) was never produced in recombinant form and biochemically characterized and it has not yet been clarified if the corresponding gene is truly expressed in mammalian cells or is only an inactive pseudogene, as annotated in the databanks.

## 3. Enzymatic Properties of Human DDX11

Analysis of the biochemical properties of a DNA helicase (in terms of DNA unwinding directionality, substrate specificity, catalytic parameters) is of paramount importance in order to understand its potential involvement in nucleic acid metabolism cellular pathways. 

Initial biochemical characterization of human DDX11 was carried out in the laboratories of Lahti [9] and Hurwitz [10]. These studies revealed that DDX11 is endowed with DNA-dependent ATPase and DNA helicase activities. DDX11 translocates on single-stranded DNA with a 5′ to 3′ directionality requiring ATP or, to a lesser extent, dATP to fuel this activity. Moreover, it was shown that DDX11 DNA strand separation requires a 5′-single-stranded region for helicase loading, since blunt-ended duplex structures do not support DNA unwinding. 

**Figure 2:**
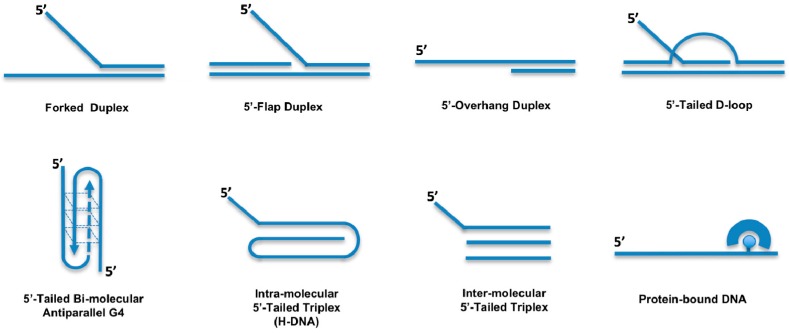
DNA substrate specificity of the human DDX11 helicase. DNA substrates unwound by human DDX11 are schematically depicted. See the text for details.

A more comprehensive analysis of the DDX11 helicase reaction requirements and DNA substrate specificity was carried out by Brosh and colleagues [11,12,13,14]. These studies revealed that DDX11 preferentially unwinds forked duplex DNA substrates with non-complementary 5′- and 3′- single-stranded arms (Figure 2). A 3′- tail having a length between 5- and 10-nt and a 5′-tail of at least 15-nt are required for the helicase to optimally melt double-stranded DNA; duplexes having blunt ends or only a 3′-tail are not unwound [11]. Moreover, the Hurwitz group reported that human DDX11 directly interacts with the Ctf18-replication factor C (RFC) complex, the proliferating cell nuclear antigen (PCNA) factor and the flap endonuclease 1 (FEN-1). The helicase activity of DDX11 was shown to be capable of displacing duplex regions up to 100 base pairs, which can be extended to 500 base pairs by replication protein A (RPA) or the Ctf18-RFC complex [10].

Double-stranded DNA molecules with a single-stranded 5′-tail are unwound, whereas substrates containing a 5′-flap structure are efficiently melted by DDX11 only if a single-stranded gap of at least 10-nt precedes the duplex region according to Farina and colleagues [10]. However, the Brosh group showed that DDX11 efficiently unwinds even a 5′ flap substrate in which only a nick resides between the 5′ flap oligonucleotide and the duplex region of the DNA substrate [11]. DDX11 is able to efficiently dismantle three-stranded D-loops with an invading 3′-end, but not Holliday junctions, which are structures similar to early and late intermediates of homologous recombination (HR) reactions, respectively (Figure 2) [11]. This substrate preference suggests a role for DDX11 in DNA repair reactions based on HR and/or in telomere metabolism, as three-stranded D-loops also resemble T-loop structures that are present at chromosomal ends [15]. DDX11 might be implicated in G-quadruplex (G4) DNA metabolism, being able to efficiently untangle bi-molecular anti-parallel G-quartets with two 5′-tails (Figure 2) [11,13]. In this respect, DDX11 was recently proposed to resolve G4 structures that can arise between sister chromatids during DNA replication at specific chromosomal locations, such as Okazaki fragments, or at homologous recombination sites [3].

Purified recombinant DNA helicases from different families were examined for their ability to unwind DNA substrates bearing a single 8,5′ cyclopurine deoxynucleoside (cPu) adduct [14]. These unusual cyclic nucleotides, which are formed as a consequence of the attack of a hydroxyl radical on the nucleotide sugar moiety, cause a perturbation of the DNA helix twist and base pair stacking and interfere with DNA replication and gene transcription [16]. The cPu adducts can be repaired by the nucleotide excision repair (NER) pathway, whereas other oxidative DNA lesions are corrected by the base excision repair (BER) mechanism [17,18]. Their accumulation is believed to accelerate tissue aging and to be predisposed to neurodegenerative diseases and cancer. While the RECQ1 and FANCJ DNA helicases are strongly inhibited by the presence of a cPu adduct in their translocating strand, DDX11 possesses an intrinsic ability to bypass and unwind DNA substrates bearing this peculiar lesion, even when they are located in the translocating strand, as revealed by in vitro enzymatic assays carried out under single turnover conditions [14]. The difference in effect of the c(Pu) lesion on the sequence-related FANCJ and DDX11 helicases suggests that they may unwind damaged DNA by distinct mechanisms. Perhaps the assembly state of DDX11 is distinct from that of FANCJ, which was shown to exist in solution as a monomer and dimer [19]. Single-molecule helicase assays should prove to be informative to examine the effect of the cPus or other DNA adducts on the DNA unwinding rate and the processivity of DDX11 and other DNA helicases. However, the precise role played by the RecQ-like and/or the Fe-S DNA helicases in the metabolic pathways for correcting and/or tolerating these oxidative DNA lesions has not yet been clarified. 

The Brosh group also analyzed the effects of highly stable alkyl phosphotriester (PTE) lesions, produced by chemical genotoxic agents, on the activity of various DNA helicases [12]. This study revealed that human DDX11, like other SF2 DNA helicases, is inhibited by a single PTE lesion located in the DNA substrate translocating strand. Conversely, a single PTE adduct in the non-translocating strand causes only a modest inhibition of DDX11 DNA melting activity. On the other hand, the SF1 DNA helicase UvrD and two hexameric ring-like replicative DNA helicases (the *Methanobacterium thermoautotrophicum* mini-chromosome maintenance-like protein and *E. coli* DnaB factor) were found to be not inhibited by a PTE lesion, irrespective of their position on the duplex DNA substrate [12].

In addition to its ability to melt duplex DNA molecules having a 5′ single-stranded tail, DDX11 was shown to be able to displace proteins bound to DNA [11]. Synthetic biotinylated oligonucleotides bound to streptavidin were used to test this peculiar enzymatic function (Figure 2). DDX11 is able to disrupt the high-affinity streptavidin:biotin interaction in a helicase protein concentration- and ATP-dependent manner. A similar protein displacement activity was also demonstrated for the Fe–S DNA helicase FANCJ, whereas the human RecQ-like DNA helicases Werner, Bloom and RECQ1 do not display this enzymatic function [20]. However, the physiological significance of the protein displacement activity has not yet been investigated, likely because no DDX11 separation-of-function mutant has yet been described that is active in duplex unwinding but, at the same time, is unable to displace DNA-bound proteins. 

More recently, Wu and colleagues reported that human DDX11 is able to untangle DNA substrates containing triple-stranded (triplex) structures [21]. This activity is ATP-dependent, has a 5′ to 3′ directionality and requires a 5′ single-stranded overhang on the third strand (Figure 2). DNA triplexes are characterized by non-canonical Hoogsteen hydrogen bonding and can be formed intra- or inter-molecularly. Intra-molecular DNA triplexes form when an appropriate sequence partially melts with one of the single strands folding back to complex with an adjacent duplex. Poly(purine/pyrimidine)-rich regions in the human genome are prone to adopting intra-molecular triplex structures that are also named hinge DNA or H-DNA [22,23]. Immunofluorescence experiments with antibodies specific for DNA triplexes revealed that these peculiar structures may exist in cells [21,24,25,26]. Interestingly, DNA triplex structures were shown to block DNA replication in vitro and in vivo and favour the formation of DNA double-stranded breaks (DSBs) in mammalian cells. Human DDX11 was found to resolve either inter- or intra-molecular DNA triplex structures [21]. Other human DNA helicases (Werner, Bloom, DHX9 and FANCJ) are able to resolve DNA triplexes, but they display a much lower catalytic efficiency on these substrates compared to DDX11 [27,28]. A comparative analysis indicated that triple-stranded DNA molecules with a 5′-overhang on the third strand represent the substrate preferred in vitro by DDX11 compared to forked and G-quadruplex DNA molecules.

The role of DDX11 in G4 and triplex DNA metabolism was investigated by studies carried out in DDX11-depleted mammalian cells, which will be described in Section 7.

## 4. Structural Features of Human DDX11

**Figure 3:**
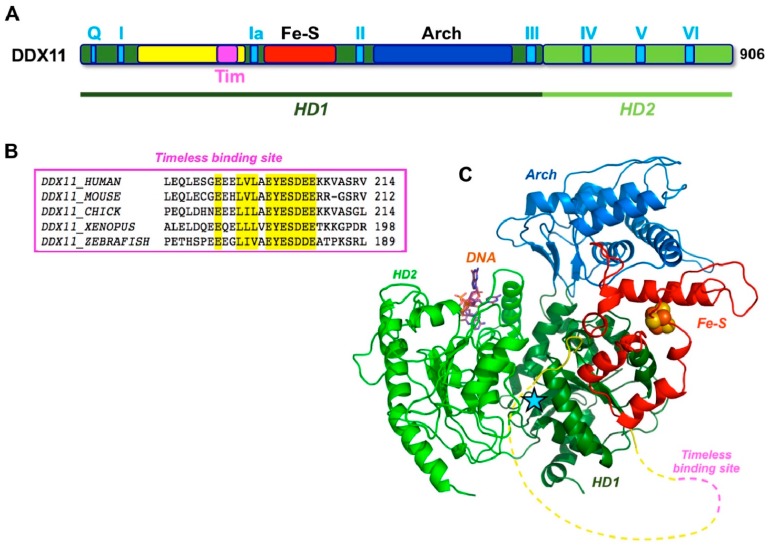
Architecture of human DDX11. (**A**) Schematic representation of the architecture of DDX11, highlighting the location of the catalytic core domains HD1 (*dark green*) and HD2 (*light green*). The other domains and motifs are shown using the same colour code as in Figure 1. (**B**) A close-up of the sequence conservation in the DDX11 region that has been shown to bind Timeless; the sequences of the human, mouse, chicken, *Xenopus* and zebrafish orthologs are shown; the residues that are conserved in all sequences are highlighted in *yellow*. (**C**) A homology model for the human DDX11 helicase, with domain colour-coded as in panel *A*; the position of the DNA is taken from the structure of an archaeal XPD (PDB ID: 4A15); a *light blue star* indicates the position of the active site. A *yellow dotted line* shows the partially disordered insertion, including the Timeless-binding region (in *magenta*).

As previously described, human DDX11 is a SF2 DNA helicase sharing sequence similarity with the XPD/Rad3 proteins from Archaea and Eukarya, *S. cerevisiae* Chl1 and the mammalian FANCJ and RTEL1 paralogues [1]. All these DNA helicases contain a Fe–S cluster. The crystallographic structure of three archaeal XPD proteins is known [29,30,31] and revealed a four-domain organization consisting of two canonical RecA folds (HD1 and HD2), forming the catalytic helicase core and two accessory domains (the Fe-S and the Arch domains), unique to this family (Figure 3). The Arch domain is positioned as a “cap” above the two RecA domains suggesting a possible role in binding the DNA substrate, whereas the exact role of the Fe-S cluster in catalysis is unknown.

Two archaeal XPD proteins were crystallised in the presence of single-stranded DNA [32,33], but only a few nucleotides were visible in the electron density and the nucleic-acid chain does not extend to the protein active site, so the structural basis of DNA binding and unwinding within this family of enzymes are still in the realm of speculation. Lower resolution cryo-electron microscopy (EM) structures are available for the human and yeast XPD within the context of the TFIIH transcription factor, but they do not shed any further light on the helicase structure and mechanism.

Multiple sequence alignments were generated with the Clustal Omega server [34] and were manually modified to account for the positioning of the experimental and predicted secondary structure elements. When the DDX11 sequences are compared with other members of the XPD/Rad3 family, a long insertion (roughly 150 amino acid long) is found in the N-terminal HD1 domain, between the motifs I and Ia, just upstream of the Fe–S domain (Figure 1). This insertion is less well conserved than the rest of the protein and contains strings of positively and negatively charged residues, suggesting that it is likely to be a partially unstructured region. A similar insertion, in the same position, is present in the FANCJ helicases, but not in the XPD and RTEL1 members of the family (Figure 1).

A three-dimensional homology model was built by integrating the results of the protein fold recognition algorithms HHPRED [35], and RaptorX [36], using as templates both the 10 Å resolution cryo-EM structure of human XPD, as found in the TFIIH complex (PDB ID: 5IVW [37]), as well as the high resolution crystallographic structure of the archaeal XPD enzyme from *Thermoplasma acidophilum* (PDB ID: 4A15 [32]). No model could be built for the putative unstructured insertion (Figure 3).

## 5. Cellular Localization of DDX11

Indirect immunofluorescence studies carried out in synchronised mammalian cells (RPE1, HeLa, Cos-7, C33a) revealed that during interphase, DDX11 has a sparse nuclear localization [38]. During prophase, DDX11 diffusely coats condensed chromatin. In the subsequent metaphase, DDX11 relocates from chromatids to spindle poles and fibres. Finally, during late telophase, DDX11 accumulates at the midbody. These localization studies were confirmed by co-staining experiments in which an anti-DDX11 antibody was used together with antibodies directed against γ-tubulin and Aurora B, proteins that specifically localize to mitotic spindle and midbody, respectively. Although these findings may suggest a role for DDX11 in spindle aster assembly and/or a function at the midbody that could be related to cytokinesis, no evidence that DDX11 may be involved in these cellular processes has been reported so far.

## 6. Role of DDX11 in Sister Chromatid Cohesion

The function of DDX11 in sister chromatid cohesion establishment appears to be conserved throughout the evolution from yeast to humans. Indeed, *CHL1* was originally identified in a screen of genes involved in chromosome maintenance and mis-segregation in budding and fission yeast [7,39,40]. Subsequent genetic studies indicated that *CHL1* gene deletion causes premature sister chromatid separation and revealed the existence of an inter-related synthetic lethal network among *CHL1*, *FEN-1*, *CTF18* and *CTF7*/*ECO1* [41,42,43,44]. All these protein factors operate at the replication fork: *FEN-1* codes for the Flap endonuclease 1, the enzyme involved in Okazaki fragment processing; *CTF18* encodes a subunit of the alternative replication factor C, responsible for loading the polymerase sliding clamp 9-1-1 when the S phase checkpoint is activated; *CTF7* corresponds to the Eco1 acetyltransferase that specifically modifies the Smc3 subunit of cohesin to ensure its stable binding to chromatin. These observations suggested that yeast Chl1 participates in the establishment of chromosomal cohesion by a mechanism proposed to take place at the replication forks in concert with lagging strand synthesis [45]. Similarly, in various human cellular systems, DDX11 down-regulation by small interfering RNAs (siRNAs) caused a profound delay in mitotic progression leading to chromosome segregation anomalies and sister chromatid cohesion defects [38,46,47]. Besides, human DDX11 was reported to directly interact with and stimulate the enzymatic activity of FEN-1 and depletion of either DDX11 or FEN-1 by siRNAs resulted in cohesion defects in human cells [10]. 

More recently, Uhlmann and colleagues. reported that budding yeast Chl1 associates with the replication factor Ctf4 on chromatin during S phase by means of a Ctf4-interacting protein (CIP) motif located in the protein C-terminal portion [48]. The Chl1 CIP box sequence is similar to those found in DNA polymerase α p180 polypeptide and Sld5 GINS subunit, both interacting with Ctf4 [49]. In the same work, it was proposed that Chl1, bound to the replication machinery through interaction with Ctf4, in turn engages in direct physical contact with the cohesin complex, promoting its acetylation by Eco1 and the establishment of cohesion. Interestingly, while the interaction of Chl1 with Ctf4 was found to be critical for sister chromatid tethering, Chl1 DNA helicase activity appeared to not be essential for this function. Conversely, budding yeast Chl1 catalytic functions were shown to be important for replication fork integrity under conditions of dNTP depletion (treatment with hydroxyurea). Therefore, a model of two separable functions (one acting in replication fork stability/DNA repair and the other one in chromosomal cohesion) was proposed for Chl1 [48]. 

However, while budding yeast Chl1 acts in concert with Ctf4 to coordinate chromosomal cohesion with DNA replication, human DDX11 was shown to work jointly with Timeless in these genome integrity maintenance processes [47,50,51]. Timeless is a component of the so-called fork-protection complex (FPC) together with Tipin. The FPC is responsible for S phase checkpoint regulation and replication machinery stabilization at difficult-to-replicate templates and replication fork barriers in unperturbed conditions [52]. In human cells, Timeless was found to co-immunoprecipitate with DDX11 and its down-regulation was seen to cause sister chromatid cohesion defects that could be rescued by ectopic over-expression of DDX11 [47]. Moreover, in either budding yeast or human cells, Chl1/DDX11 interacts with the cohesin complex on chromatin [47,53]. More recently, Cortone and colleagues demonstrated that human DDX11 directly interacts with Timeless through a conserved peptide (the E^200^-Y^201^-E^202^ motif, located between helicase box I and Ia) (Figure 3) and this interaction is critical for a stable association of cohesin to the replication forks and for chromosomal cohesion in S, G2 and M phase. In the same study, DDX11 was found to localize at sites of DNA synthesis by the in situ analysis of protein interactions at DNA replication forks (SIRF) technique [51]. Moreover, in agreement with what was reported for budding yeast Chl1 [48], the DNA helicase activity of human DDX11 was found not to be essential for sister chromatid tethering, since DDX11 helicase-dead mutants could partially rescue cohesion defects in DDX11-depleted HeLa cells [51]. On the other hand, in chicken DT40 cells, the DDX11 helicase activity was found to make a more important contribution to cohesion establishment [53]. It is not clear if this may reflect unique aspects of the chromosomal cohesion mechanism in avian DT40 versus yeast or human cells.

Work carried out by the Huarte group revealed that the role played by human DDX11 in sister chromatid cohesion might be regulated by a long noncoding RNA, CONCR (cohesion regulator non-coding RNA), whose transcription is activated by Myc [54]. CONCR, previously annotated as DDX11 antisense RNA 1 (DDX11-AS1), is a divergent non-overlapping transcript of the *DDX11* gene. CONCR was found to be up-regulated in multiple cancer types, when comparing tumour specimens with healthy tissue-paired samples. CONCR expression is down-regulated in p53 proficient cells; whereas, in a *p53*-/- genetic background (such as in *p53*-/- HCT116 and A549 cell lines), its expression is increased. CONCR is ubiquitously expressed in human cell lines derived from different tissues and is localised in the nucleus, as revealed by RNA fluorescence in situ hybridization (FISH) analyses. The expression of CONCR is tightly regulated across the cell cycle and is needed for efficient G1/S progression and DNA replication. Inactivation of CONCR causes a severe defect in sister chromatid cohesion, a phenotype that can be efficiently rescued by over-expressing DDX11. However, CONCR depletion does not affect the DDX11 RNA and protein level, suggesting that it may influence sister chromatid cohesion by directly interacting with DDX11. Interestingly, CONCR and DDX11 co-fractionate during chromatin purification from cell extracts by CsCl density-gradient centrifugation. In the absence of CONCR, DDX11 was shifted from the chromatin to the soluble fraction of the cell extracts. This suggests that DDX11 association to chromatin is dependent on CONCR. CONCR was found to enhance the ATPase activity of purified recombinant DDX11. However, it was not clarified if this effect is specific and if CONCR is also able to stimulate the DDX11 DNA helicase activity, either in vitro or in vivo [54].

Two main models were proposed to explain the role played by Chl1/DDX11 in coupling sister chromatid cohesion with DNA replication. In the first model, an important function is assigned to the Chl1/DDX11 DNA helicase activity that might be required to resolve peculiar DNA secondary structures (triplex- or G-quadruplex-containing DNA, stable hairpins and so on) arising at the replication forks, mainly on the lagging strand. This activity would ensure a timely and efficient maturation of the Okazaki fragments and a smooth interaction of the advancing replisomes with the cohesin rings bound to the DNA template [3,45]. 

In the second model, proposed by Uhlmann and colleagues on the basis of genetic and biochemical studies in budding yeast, Chl1 was hypothesized to have a scaffolding function at the replication fork, since its DNA helicase activity was found to be dispensable for sister chromatid tethering [48]. According to this proposal, Chl1, recruited to the replication fork by Ctf4, would position cohesin in a conformation able to capture the two sister DNA molecules: first, the duplicated leading strand is topologically entrapped inside the cohesin ring and, then, a single-stranded region on the lagging strand is bound (the so-called “second single-stranded DNA capture” mechanism) followed by Smc3 acetylation by Eco1 [55]. The results of a very recent comprehensive analysis of the contribution of many human DNA replication factors to sister chromatid cohesion seem to be in agreement with the latter model [56]. This work revealed that depletion of FEN-1 and DNA ligase 1, enzymes involved in Okazaki fragment processing, does not cause overt cohesion defects in HeLa cells. The same phenotype was observed in cells, where Chaf1, a histone deposition factor, was downregulated. These findings suggest that cohesion establishment takes place independently and prior to Okazaki fragment maturation and chromatin assembly [56].

The role played by Chl1/DDX11 and the contribution of its DNA helicase activity to sister chromatid cohesion establishment in yeasts and metazoans needs to be further explored.

## 7. DDX11 Functions in DNA Repair and Replication Fork Stabilization

The involvement of Chl1/DDX11 in DNA repair mechanisms appears to be evolutionarily conserved. Budding yeast Chl1 was reported to preserve genome integrity upon DNA damage induced by treatment with methylmethane sulfonate (MMS) or ultraviolet (UV) rays [57,58]. An interesting study performed by Inoue and colleagues indicated that mammalian DDX11 might participate in a DNA repair process based on HR between sister chromatids, since its depletion was found to reduce the level of sister chromatid exchange (SCE) induced by the mutagen 4-nitroquinoline1-oxide in HeLa cells [46]. The participation of human DDX11 in DNA repair pathways was underlined by the finding that DDX11-knockdown HeLa cells are highly sensitive to cisplatinum and bleomycin, a radio-mimetic compound that induces the formation of DSBs [59]. 

Very recently, the Branzei group analyzed the role of DDX11 in DNA repair in DT40 chicken cells. Using this genetically amenable system, the functional consequences of *DDX11* gene knockout were examined, even in combinations with genetic ablation of other DNA repair genes, including *FANCJ*, *FANCC*, *BRCA2*, *RAD17*, and *PRIMPOL* [60]. Chicken DDX11 was found to promote the repair of DNA bulky lesions (as the ones induced by MMS) by HR and to facilitate trans-lesion synthesis through abasic sites in concert with the 9-1-1 checkpoint clamp and its loader, Rad17, mainly in a post-replicative manner. Moreover, avian DDX11 was found to be involved in the repair of DNA inter-strand cross-links (ICLs) acting in a pathway that appears to be genetically distinct and subsidiary to the Fanconi anemia one. Branzei and colleagues proposed a model, in which DDX11 unwinds the 3′-end of the newly-replicated strand stalled in proximity of the DNA lesion, while the 9-1-1 alternative polymerase clamp favours the formation of a Rad51 nucleofilament on the postreplicative single-stranded gap; thereafter, strand invasion and maturation of an HR intermediate (a double-Holliday junction) leads to repair of the DNA lesion. Alternatively, if the 9-1-1 factor is absent, DDX11 might promote recruitment of a specialized trans-lesion DNA polymerase at the damaged site to by-pass the lesion [60]. In this same study, avian DDX11 was demonstrated to promote immuno-globulin (Ig) diversity in chicken B cells by increasing mutation and gene conversion frequency at programmed abasic sites in the Ig-variable gene locus. Abasic sites, which are produced by the combined action of a cytidine-deaminase and an uracil-glycosylase, are fixed in B cells by gene conversion events involving the upstream pseudogenes or by mutagenic trans-lesion synthesis, giving rise to immunoglobulin diversification. In avian B cells, where *DDX11* and/or *RAD17* are knocked-out, both these DNA repair mechanisms (and the consequent antibody diversification) are reduced [60].

In a study carried out in the Brosh laboratory, the sensitivity of helicase-depleted human cell lines to Telomestatin, a G4 DNA-binder, was analyzed [13]. In these experiments, formation of γH2AX foci, detected by indirect immunofluorescence, was used as a readout of DNA damage induced by treating cells with Telomestatin. Results of this analysis indicated that U2OS cells, where DDX11 was downregulated by siRNAs (along with *XPD*-/- cells derived from a *Xeroderma pigmentosum* patient), were resistant to Telomestatin treatment and did not display an increased number of γH2AX foci compared to control cells. In contrast, FANCJ-depleted cells showed a clear increase of DNA damage *foci* following Telomestatin treatment compared to control cells. In parallel experiments, either DDX11- or FANCJ-depleted U2OS cells were found to be highly sensitive to the DNA cross-linking agent mitomycin *C* (MMC), as previously reported [61,62]. These findings are in agreement with biochemical analyses revealing that FANCJ is unique among the other tested DNA helicases (DDX11, XPD) in the ability to resolve unimolecular G-quadruplexes [13]. These G4 structures are likely to form in transiently melted G-rich genomic sites, especially on single-stranded regions of the lagging strand during DNA replication. In contrast, DDX11 is almost completely unable to untangle unimolecular G-quartet DNA structures, as described in Section 3. 

In enzymatic assays carried out, in vitro human DDX11 was found to be extremely active on DNA substrates containing triplex DNA structures [21]. The physiological relevance of this activity was investigated in HeLa cells, where expression of DDX11 was down-regulated with a short hairpin shRNA produced by a retrovirus-based vector. Cells were treated with a triplex DNA-stabilizing agent (benzoquinoquinoxaline, BQQ) and indirect immunofluorescence with specific antibodies was used to visualise triplex DNA and γH2AX *foci*, as DNA damage markers. Treatment with BBQ caused a remarkable increase of triplex DNA structures and DSBs in DDX11-depleted cells compared to control cells. The effect of FANCJ loss in triplex DNA metabolism was also examined using FANCJ-deficient and -proficient isogenic cell lines derived from a Fanconi anemia proband [21]. Occurrence of triplex DNA and γH2AX *foci* was not significantly changed between these cell lines, treated or not with BBQ. However, in *FANCJ*-/- cells, significantly lower levels of triplex DNA *foci* were detected compared to DDX11-downregulated cells. These results suggest that DDX11 has a prominent role in defending genome stability against the formation of triple-stranded DNA structures compared to FANCJ. However, other human DNA helicases (such as the Werner and Bloom proteins and the RNA/DNA helicase DHX9) were reported to resolve DNA triplexes in vitro [26,27,63]. 

A role for DDX11 in preserving replication fork integrity under stressful conditions was highlighted in a study performed in collaboration by the Pisani and Brosh groups, where it was reported that DDX11 and Timeless physically and functionally interact and act in concert to assist replisome smooth progression in hydroxyurea-treated HeLa cells [50]. DNA fibre track assays revealed that depletion of either DDX11 or Timeless reduced replication fork progression compared to control cells following hydroxyurea treatment; no additive effect was observed by co-depletion of both proteins. DDX11 and Timeless were found to be epistatic in promoting efficient resumption of stalled DNA replication forks after a prolonged treatment of HeLa cells with hydroxyurea. Timeless was shown to establish a direct physical interaction with DDX11 and to stimulate its DNA helicase activity on diverse DNA substrates, including forked DNA substrates, bimolecular anti-parallel G4 and three-stranded D-loop structures (Figure 2). Electrophoretic mobility shift assays revealed that Timeless enhances DDX11 binding to DNA. This result suggests that the observed stimulation of the DNA helicase activity derives from an improved ability of DDX11 to interact with the nucleic acid substrate in the presence of Timeless [50]. However, the precise molecular mechanism by which DDX11 and Timeless counteract replication stress needs to be further investigated.

Intriguingly, two independent research groups reported that Timeless directly interacts with the poly(ADP-ribose) polymerase 1 (PARP-1); both proteins accumulate in a rapid and transient way on chromatin at laser-induced DNA damage sites and cooperate in the same HR–mediated DNA repair pathway [64,65]. It would be interesting to analyze if DDX11 and cohesin are recruited to damaged DNA sites in a PARP-1:Timeless-dependent manner. In this context, it is of note that DDX11-deficient cells, derived from a WABS patient, were found to be highly sensitive to PARP inhibitors [66]. 

It is not clear if roles in DNA repair and in sister chromatid cohesion establishment represent separable functions of metazoan DDX11, as proposed for Chl1, the yeast orthologous protein [48]. The identification of DDX11 separation-of-function mutants, proficient in DNA repair but unable to promote chromosomal cohesion and/or vice versa, will help address this issue.

## 8. DDX11 and Chromatin Structure

Mammalian DDX11 was proposed to have a role in heterochromatin organization [67]. DDX11 is responsible for the correct recruitment of heterochromatin protein-1, isoform α, (HP1α) at chromosomal pericentric and telomeric sites. Mammalian HP1 proteins (isoforms α, β and γ) are involved in the assembly of higher-order chromatin structure and epigenetic inheritance. At constitutive heterochromatin, HP1α specifically binds Lys9-methylated histone H3 (H3K9-me3) through its chromo-domain (CD) and at the same time, by means of its chromo-shadow domain (CSD), it recruits various epigenetic modifiers, including SUV39H1 and SUV39H2 (the methyl-transferases of histone H3) and DNMT1 and DNMT3a (the DNA methyl-transferases acting at CpG sequences). In DDX11-depleted HeLa cells, constitutive heterochromatin exhibits disperse localization with disrupted centromere clustering, while in control cells it is discretely localized at perinuclear and perinucleolar regions. Furthermore, DDX11-downregulation causes reduced binding of HP1α and decreases levels of H3K9-me3 at pericentric and telomeric regions. Collectively, these findings suggest that DDX11 has a role in heterochromatin formation and global nuclear organization, raising the possibility that it could also affect the regulation of gene expression/accessibility [67].

It was reported that DDX11 is localised to nucleoli in mammalian cells during interphase and acts as a positive regulator of ribosomal RNA gene transcription [68]. DDX11 down-regulation caused reduced association of the upstream binding factor (UBF) and a subunit of RNA polymerase I (named RPA 194) to the promoter of the 47S ribosomal RNA gene. In DDX11-depleted cells phosphorylation and acetylation of UBF, modifications required for transcriptional activation of the ribosomal RNA genes, are strikingly reduced in response to serum stimulation. Consistently, DDX11 knockdown changed the epigenetic *status* of chromatin regions containing the ribosomal RNA genes from euchromatic to more heterochromatic (reduced level of trimethylated Lys4 in histone H3, H3K4me3, along with an increased level of trimethylated Lys9 in histone H3, H3K9me3). It was proposed that dysfunction in ribosome biogenesis caused by DDX11 loss results in specific developmental defects, as the ones observed in WABS-affected individuals (see Section 10). This hypothesis was corroborated by the observation that down-regulation of the zebrafish DDX11 ortholog by morpholinos results in a reduced level of ribosomal RNA precursors and epigenetic modifications at the corresponding ribosomal RNA gene *loci*, concomitant with anomalies similar to clinical manifestations of WABS (such as growth retardation and vertebral and craniofacial malformations).

Collectively, all these studies suggest that DDX11 could modulate the epigenetic status of various chromatin regions affecting the transcription of genes located therein.

## 9. DDX11 and Cancer

DNA helicases involved in genome stability maintenance have a complex role in oncogenesis: Whereas inactivation of these enzymes may be predisposed to cancer onset, their expression is up-regulated in many tumour cells, likely reflecting the need to counteract the increased level of replicative stress in rapidly-proliferating cells. Thus, DNA helicases may become important factors for the survival and proliferation of tumours and for the resistance to DNA-damaging agents used in chemotherapy [2]. Searches through online catalogues of mutations in cancer reveal that the *DDX11* gene is mutated, highly up-regulated and targeted for copy number amplification in many diverse tumour tissues. More than 20% of the *DDX11* recorded cancer mutations are clustered and missense, suggesting that it may function as an oncogene, more than an onco-suppressor, according to the 20:20 rule [69,70].

In agreement with these findings, a potential crucial role was proposed for DDX11 in the survival of advanced melanomas by the Becker group [71]. To identify genes up-regulated in the transition from non-invasive melanoma in situ (MIS) to radial growth phase (RGP) melanoma, the first stage of invasive melanoma, whole genome expression analysis was carried out on RNA samples isolated from archived fixed tissues representing these two stages of the disease. This analysis revealed that the *DDX11* gene was strikingly (eightfold) up-regulated in RGP- versus MIS-derived samples. Moreover, immunohistochemistry studies revealed higher expression of DDX11 in advanced melanoma and melanoma-infiltrated lymph nodes compared to epidermal melanocytes of normal skin, atypical nevocytes and MIS cells. DDX11 downregulation by siRNAs in cells derived from metastatic growth phase (MPG) melanoma had the following consequences: rapid and dramatic morphology alteration, sister chromatid cohesion defects, chromosome fragmentation, inhibition of cell proliferation, and massive apoptosis. These results prompted Becker and colleagues to propose DDX11 as an important target to devise novel pharmacological strategies to cure advanced melanoma refractory to conventional chemo- and/or radio-therapy [71]. 

Moreover, it was reported that in the colorectal cancer cell lines HCT116 and DLD1 and in ALK-positive anaplastic large cell lymphoma (ALCL), an aggressive form of non-Hodgkin lymphoma, DDX11 protein expression level is reduced, leading to genomic instability and chromatid cohesion defects [72,73,74]. In mammalian cells, *DDX11* gene expression was found to be regulated by JunB in G2/M [74]. JunB is a component of the activator protein 1 (AP-1) transcription factor, whose expression level is tightly regulated during the cell cycle. JunB levels decrease by mid-to-late G2 due to proteasome-mediated degradation. This process is under the control of the protein kinase GSK3β and the FBXW7 factor. Following phosphorylation by GSK3β at an evolutionarily conserved phospho-degron, JunB is bound by FBXW7, a substrate-adaptor of the SCF ubiquitin ligase, and subjected to proteasome-mediated degradation. In the above mentioned HCT116 and DLD colorectal cancer cell lines, JunB strongly accumulates during prometaphase, leading to *DDX11* gene repression. JunB is up-regulated in ALK-positive ALCL cells, where the GSK3β kinase is inactivated by the action of the oncogenic kinase AKT [74]. Overall, these findings revealed that transcriptional regulation of the *DDX11* gene takes place through the GSK3β-FBXW7-JunB axis in mammalian cells and pharmacological or genetic inactivation of this pathway gives rise to JunB accumulation in G2/M and consequent transcriptional repression of the *DDX11* gene. In turn, loss of the DDX11 protein leads to sister chromatid cohesion defects, mis-segregation, multiple chromosomal abnormalities, and genomic instability [74]. 

In this context, it is of interest that a genome-wide siRNA screen in DDX11-depleted cells derived from a WABS patient revealed that these cells do not tolerate partial loss of individual subunits of the anaphase promoting complex (APC/C) [75]. The APC/C, a large complex of 11-13 subunits, is an E3 ubiquitin ligase that modifies target cell cycle proteins to be degraded by the 26S proteasome triggering the transition from metaphase to anaphase. Three major substrates of the APC/C are: Securin, which blocks Separase, the protease that specifically cleaves cohesin Rad21 subunit; cyclin B1, which activates the mitotic kinase Cdk1; and Geminin, which binds the replication factor Cdt1 inhibiting pre-replication complex assembly. When the chromosomes are properly attached to the mitotic spindle and their centromeres are under tension, the spindle attachment checkpoint (SAC) is silenced and the APC/C is activated leading to degradation of Securin, cyclin B and Geminin. Then, cohesin cleavage by Separase allows chromatid migration opposite to spindle poles; inactivation of Cdk1 initiates cytokinesis and mitotic exit; Geminin degradation prepares cells for the subsequent DNA replication. To explain the observed synthetic lethality interactions of DDX11 with the APC/C subunits, Wolthuis and colleagues proposed a model based on the so-called “cohesion fatigue”, a phenomenon typically occurring in cells with cohesion anomalies. Cohesion fatigue is the unscheduled chromatid separation that takes place during prolonged metaphase. In cells with impaired chromosomal cohesion (such as the ones derived from a WABS patient), partial inactivation of the APC/C can be detrimental, as it can give rise to a mitotic arrest. In fact, in these cells, reduction of spindle tension at centromeres, due to cohesion fatigue, keeps the SAC in active form leading to a full inhibition of the APC/C and a metaphase arrest. Thus, additional sister chromatid pairs may undergo cohesion fatigue, further blocking cells in mitosis. Eventually, this will cause cell death or aberrant exit from mitosis with generation of aneuploidy [75]. The synthetic lethal interaction between DDX11 and APC/C subunits could be exploited in novel therapeutic strategies for those cancers, where DDX11 expression is down-regulated, as described above.

Parish and colleagues revealed an essential role for DDX11 in the establishment and maintenance of human papilloma virus (HPV), which is the causative agent of cervical cancer and other cancers of genitals and oral cavity [76]. HPVs are a large family of double-stranded DNA viruses that infect cutaneous and mucosal epithelia. During the HPV life cycle, the viral genome is maintained as an autonomous, multi-copy circular double-stranded DNA plasmid. Its integration in the host genome is considered a risk factor for cancer progression. The HPV DNA does not contain a canonical centromeric region and therefore cannot assemble a kinetochore-like structure that could be used to facilitate its segregation during mitosis. Episomal HPV genome replication and separation into daughter cells is regulated by the E2 viral protein with a mechanism yet unresolved. E2 binds specific sequences within the viral genome through a C-terminal DNA binding domain (DBD), while, at the same time, its N-terminal transactivation domain (TD) engages with host cell chromatin proteins. Parish and co-workers found that E2 associates with DDX11 during S phase and during very early stages of mitosis and proposed that this association is important for viral genomes chromatin-tethering during DNA replication, a mechanism essential for HPV persistence and productive infection [76,77,78]. This proposal was based on the results of co-immuno-precipitation assays in cell extracts and fluorescence resonance energy transfer-based experiments in live cells synchronised at specific stages of the cell cycle [77]. The binding site for HPV E2 was mapped between amino acids 130 and 214 of the DDX11 polypeptide chain, although a direct interaction between the two proteins was not formally proved [78]. Of note, the E2 binding site lies within the DDX11 N-terminal insertion between helicase boxes I and Ia (residues 65-225), which also contains the E^200^-Y^201^-E^202^ motif critical for the interaction with Timeless (Figure 3) [51]. Therefore, it would be of interest to examine if DDX11 binding sites for HPV E2 and Timeless are distinct or overlap and if Timeless influences the DDX11:E2 protein interaction in any way during the HPV life cycle. This would expand the repertoire of DNA tumour viruses whose genome replication and maintenance is found to be dependent on Timeless [79,80]. 

## 10. Warsaw Breakage Syndrome, a Cohesinopathy

Warsaw breakage syndrome (WABS) is a rare autosomal recessive disease due to homozygous mutations in the gene coding for DDX11 [62,81,82,83,84]. WABS patients display several clinical features, including severe pre- and post-natal growth retardation, microcephaly, sensorineural hearing loss, cochlear anomalies, facial dysmorphy, abnormal skin pigmentation, cardiac defects, and intellectual disability. 

Analysis of cultured T lymphocytes and immortalized B lymphoblasts from WABS patients showed increased MMC-induced chromosomal breakage, a phenotype reminiscent of Fanconi anemia (FA), a genetic disorder characterized by progressive bone marrow failure, congenital abnormalities and cancer predisposition. Cells derived from WABS patients show sister chromatid cohesion anomalies. Specifically, the chromosomes are distinguished by a “railroad” appearance, presumably due to premature centromere division, as well as a high proportion of total premature chromatid separation defects, in particular, after treatment with DNA cross-linking agents. Other reports also attest to a role of DDX11 and its *S. cerevisiae* ortholog, Chl1, in sister chromatid cohesion establishment [38,40,44]. These cohesion defects resemble the ones observed in metaphase lymphoblasts from individuals affected by Roberts syndrome (RBS), another rare autosomal recessive disease due to mutations in the *ESCO2* gene [85]. Esco2 is a putative acetyltransferase required for chromosomal cohesion establishment during S phase and is one of the two human orthologs of budding yeast Eco1 [86,87]. Both WABS and RBS are classified as cohesinopathies, genetic diseases caused by mutations in genes involved in cohesion. Other cohesinopathies include Cornelia de Lange syndrome (CdLS), caused by mutations in genes encoding cohesin structural components (*SMC1A*, *SMC3* and *RAD21*) and regulators (*NIPBL* and *HDAC8*) and Chronic Atrial and Intestinal Dysrhythmia (CAID) syndrome, linked to mutations in the *SGOL1* gene encoding Shugoshin [88,89,90]. Both WABS and RBS are very rare diseases with a prevalence estimated to be less than 1/1 × 10^6^. However, it is likely that the incidence of WABS is underestimated, because of a diagnostic overlap with FA [91]. 

**Figure 4:**
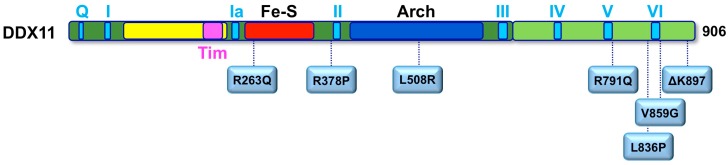
Missense mutations found in Warsaw breakage syndrome (WABS) patients. Schematic representation on the human DDX11 polypeptide chain (colour-coded as in Figure 3A), showing the location of the amino-acid substitutions described in individuals affected by WABS. See text for details.

To date, a total of 12 WABS probands were described in the literature with different gene mutations (Figure 4). The first case was reported in 2010 by de Winter and colleagues in a boy from Warsaw (Poland), inspiring the disease name [62]. Sequence analysis of the patient genomic DNA uncovered two mutations in the *DDX11* gene: a splice-site mutation in intron 22 of the maternal *DDX11* gene allele (leading to deletion of the last 10 bps of exon 22 and to a premature stop codon in exon 23), and a 3-bp deletion in exon 22 of the paternal allele (leading to deletion of the triplet coding for the highly conserved residue K897). The resulting DDX11-ΔK897 mutant protein is catalytically inactive and unstable. Later, three affected siblings in a Lebanese consanguineous family were identified as bearing a novel *DDX11* bi-allelic homozygous mutation resulting in substitution of a conserved Arginine with Glutamine (R263Q) in the protein Fe–S motif [81], which severely impairs the DNA helicase activity. These WABS probands show severe intellectual disability in addition to growth defects, deafness and dysmorphic facial features. The third case of WABS was described in a British girl, born from non-consanguineous parents [82]. The patient bears two heterozygous mutations introducing frame-shifts in the *DDX11* open reading frame, and, in addition to other known features, she presented a chronic skin rash with telangiectasia on her legs as a novel clinical manifestation. In 2017, two sisters were diagnosed with WABS: they were reported to have limb malformations in addition to the typical clinical features [83]. Both sisters were found to bear *DDX11* mutated alleles: a maternally inherited missense mutation (L508R within the Arch domain) and a paternally derived variant predicted to affect splicing. Very recently, five novel additional WABS probands were described with five novel bi-allelic *DDX11* gene variants [84]. Two of these novel mutations introduce a frame-shift in the DDX11 coding sequence, whereas two of them are missense variants that map in the vicinity of the conserved helicase box V. The fifth novel identified mutation is homozygous and causes an amino acidic change (R378P) that was demonstrated to tremendously affect DDX11 protein stability. More recently, a novel mutation (L836P; Figure 4) was identified in two Italian young sisters, showing a mild phenotype with no major physical or intellectual disabilities (Faletra and co-workers, manuscript in preparation).

While sister chromatid cohesion defects were observed in all the patients described so far, two of these novel WABS patients do not display drug-induced elevated chromosomal breakage. Thus, while diagnostic clinical symptoms of WABS (microcephaly, growth retardation, cochlear anomalies and chromosomal cohesion defects) are observed in all cases reported in the literature, the chromosomal breakage phenotype is not universally present. In view of these findings, it was proposed to rename this disease “Warsaw syndrome”, eliminating reference to the chromosomal breakage phenotype [84]. 

The observed phenotypic differences among WABS patients may be due to the different effects of the *DDX11* gene mutations and/or to different genetic background of the affected individuals. The ethiopathogenesis of WABS has not yet been deciphered as the cellular functions of DDX11 are not fully understood. However, the partial overlap of clinical manifestations (growth retardation, microcephaly, intellectual disability) with other cohesinopathies (CdLS and RBS) suggests that all these diseases share common developmental defects due to altered transcription profiles during embryonic development. This is consistent with the evidence that cohesin and its regulators play a role in stabilizing chromatin loops, through which developmental gene transcription programs are executed. In this context, it is interesting to point out that either DDX11 or Esco2 were reported to play an important function in chromosome architecture maintenance and their depletion in mammalian cells causes chromosome condensation defects in addition to sister chromatid cohesion anomalies, as described in Section 8. It was proposed that Esco2 could be responsible for recruiting chromatin modifiers (such as histone H3 methyltransferases and demethylases) affecting gene expression [92]. Moreover, DDX11 was believed to contribute to heterochromatin formation by targeting HP1α factor to proper sites in pericentric regions and at telomeres in DDX11-depleted HeLa and in DDX11 knockout mouse embryo-derived cells [67]. Based on these findings, it was postulated that all cohesinopathies are due to anomalies in cellular pathways involving cohesin and cohesin-related factors that affect gene expression/accessibility during embryogenesis, causing the observed multi-spectrum developmental anomalies. Our recent finding that DDX11 plays a critical role in cohesin loading onto chromatin in HeLa cells [51] and the previous report that Chl1 is critical for deposition of the loader complex subunit Scc2 to chromatin in budding yeast [93] extends the validity of the transcription-based cohesinopathy model to include even WABS [94]. A different paradigm for rationalizing the ethiology of different cohesinopathies envisages that these hereditary disorders are caused by altered gene expression due to defective translational mechanisms [95,96]. A peculiar feature of chromosomes derived from RBS patients is the presence of "puffing" chromatin at centromeres and at nucleolar organizing regions [85]. In RBS fibroblasts production of ribosomal RNA and the number of actively translating ribosomes is noticeably reduced compared to control cells, suggesting that a global reduction of the translational efficiency is an additional defect associated with this cohesinopathy [97]. Moreover, mutations in genes coding for cohesin subunits were found to be associated with nucleolar dysfunction, indicating that maintenance of cohesion is critical for ribosomal RNA synthesis and ribosome biogenesis [98,99]. In addition, it was recently found that DDX11 localizes at the nucleolus in HeLa cells and preferentially binds to hypomethylated active ribosomal DNA gene *loci*, where it interacts with the RNA polymerase I transcriptional machinery. DDX11 down-regulation caused suppression of ribosomal RNA synthesis, inhibiting cell proliferation [68]. Finally, in DDX11-knockdown zebrafish embryos, the epigenetic status of ribosomal DNA gene cluster was changed from euchromatic to heterochromatic and recruitment of RNA polymerase I to these *loci* was significantly decreased along with pre-RNA levels. These results suggest that the developmental defects induced by DDX11 loss in zebrafish are at least partially associated with nucleolar dysfunction [68]. 

## 11. Conclusions

**Figure 5:**
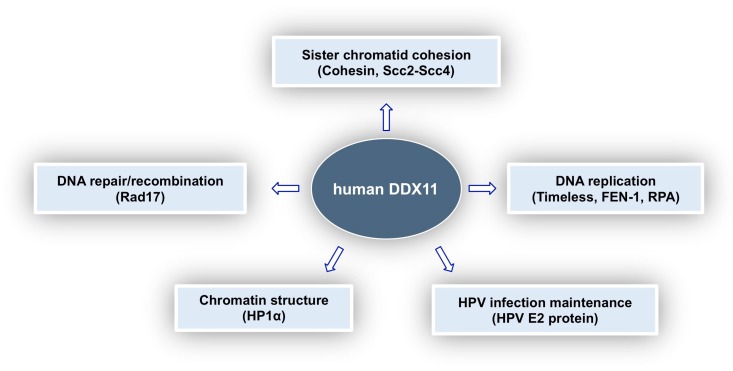
Cellular roles of human DDX11 and relevant protein interactions. DDX11 was shown to play a role in various pathways. Interaction of DDX11 with the indicated protein partners was proposed to be important for these cellular functions, as described in the text.

In this review, we tried to provide a comprehensive picture of what is known about the multi-faceted roles played by DDX11 in genome stability maintenance. As summarised in Figure 5, DDX11 was reported to interact with a number of different proteins involved in DNA replication, DNA damage response, sister chromatid cohesion and chromatin architecture and is thought to be a key player in all these important cellular pathways. Discovery in 2010 of the linkage between *DDX11* gene mutations and Warsaw breakage syndrome, a rare genetic disease, highlighted for the first time its medical relevance [62]. Furthermore, DDX11 role as a genome caretaker opens up the possibility to target it in personalised treatment of cancers, where synthetically lethal genome stability pathways are altered. Much has still to be learned in order to understand the molecular and cellular functions of DDX11 and to devise specific therapeutic strategies.
